# Is arch form influenced by sagittal molar relationship or Bolton tooth-size discrepancy?

**DOI:** 10.1186/s12903-015-0062-2

**Published:** 2015-06-26

**Authors:** Abdullah M. Aldrees, Abdulmajeed M. Al-Shujaa, Mohammad A. Alqahtani, Ali S. Aljhani

**Affiliations:** 1 Department of Pediatric Dentistry and Orthodontics, College of Dentistry, King Saud University, P.O. Box 60169–38, Riyadh, 11545 Saudi Arabia; 2 Resident, Department of Dentistry, King Fahad Medical City, Riyadh, Saudi Arabia; 3 Resident, Advanced Education in General Dentistry Program, School of Dental Medicine, State University of New York at Buffalo, Buffalo, NY USA; 4 Division of Orthodontics, College of Dentistry, King Saud bin Abdulaziz University For Health Sciences, Consultant Orthodontist, National Guard Health Affairs, Riyadh, Saudi Arabia

**Keywords:** Bolton discrepancy, Arch form, Angle class

## Abstract

**Background:**

Orthodontic patients show high prevalence of tooth-size discrepancy. This study investigates the possible association between arch form, clinically significant tooth-size discrepancy, and sagittal molar relationship.

**Methods:**

Pretreatment orthodontic casts of 230 Saudi patients were classified into one of three arch form types (tapered, ovoid, and square) using digitally scanned images of the mandibular arches. Bolton ratio was calculated, sagittal molar relationship was defined according to Angle classification, and correlations were analyzed using ANOVA, chi-square, and t-tests.

**Results:**

No single arch form was significantly more common than the others. Furthermore, no association was observed between the presence of significant Bolton discrepancy and the sagittal molar relationship or arch form. Overall Bolton discrepancy is significantly more prevalent in males.

**Conclusions:**

Arch form in a Saudi patient group is independent of gender, sagittal molar relationship, and Bolton discrepancy.

## Background

Orthodontic diagnosis and treatment planning require properly trimmed study casts in order to analyze dental relationships. One of these measurements is tooth-size discrepancy, which is defined as disproportionate sizing of opposing teeth [1]. Bolton overall and anterior ratios between opposing teeth should be normal to ensure ideal interdigitation, overbite, and overjet [2].

Many investigators evaluated the effect of tooth-size discrepancy on occlusion among different malocclusion groups, sexes, and ethnicities. Nie and Lin showed that tooth-size discrepancy was highly prevalent in Class III and uncommon in Class II [3]. Araujo and Souki also reported higher prevalence of tooth-size discrepancy in individuals with Class III than with Class I malocclusion [4]. This trend was also reported in a southern Chinese population and a Saudi population [5, 6]. Individuals with malocclusion present with significantly higher tooth-size ratios than those with untreated normal occlusions [7].

Lavelle found that overall and anterior ratios are higher among males than females, regardless of race [8]. Santoro *et al.* found that male crown measurements are slightly larger and show higher variability than female measurements, which in turn demonstrates differences in tooth-size discrepancy (TSD) between sexes [9]. Uysal *et al.* showed significant sex differences for overall ratio among normal occlusion subjects [7]. However, Sameshima [10], Araujo and Souki [4], Akyalcin *et al.* [11], Basaran *et al.* [12], Nie and Lin [3], Al-Tamimi and Hashim [13], and Endo *et al.* [14] reported no significance sex difference in tooth-size ratios.

Ethnicity is a factor in tooth-size ratios. Individuals of African ethnic background have been reported to have larger teeth than Caucasian individuals [2]. Dominican Americans have been reported to have tooth sizes similar to African Americans but larger than European Americans [9]. Johe *et al.* found that African American subjects had higher prevalence of clinically significant anterior tooth-size discrepancies than did Caucasians and Hispanics; and discrepancies among Hispanic patients were more likely due to mandibular anterior excess [15]. The mathematical tooth-size ratios introduced by Bolton are intended to serve as a useful guide in diagnosis and treatment planning [16]. However, Smith *et al.* reported that Bolton ratios apply to white women only but are not applicable to white men, blacks, or Hispanics [2]. Other studies showed that Bolton values are not applicable to Turkish or Japanese populations [14, 17]. However, Al-Tamimi and Hashim found no differences between Bolton ratios and the tooth-size ratios of their Saudi sample.

Preformed archwires are commonly used in orthodontic practice [18]. Several authors have pointed to the importance of classifying the patient’s arch form for selection of appropriate preformed archwire to achieve stability of the therapeutic results [19]. Felton *et al.* investigated the possibility of an ideal orthodontic arch form that might be identified for treated and untreated individuals, but found no a specific arch form [20]. Raberin *et al.* found five predominant mandibular dental arch forms (narrow, wide, mid, pointed and flat) in their sample of French individuals with normal occlusion [21]. Nojima *et al.* compared morphological difference between Caucasian and Japanese mandibular arches and concluded that no single arch form is specific to any Angle classification or ethnic group [22]. Kook *et al.,* Gafni *et al.,* and Bayome *et al.* followed the method prescribed by Nojima *et al.* to determine the arch forms in different populations [23–25]. Taner *et al.* evaluated longitudinal arch width and form and concluded that maxillary arch forms were mostly tapered, and that mandibular arches were tapered and narrow-tapered [26].

Trivino *et al.* identified 23 mandibular arch forms in a Brazilian group and concluded that a single arch form cannot represent the normal dental arch [27].

Oda *et al.* found that preformed archwires were significantly narrower than normal dental arches [28]. Subjective classification of dental-arch shape and objective analysis via arch-width measurements were found to be correlated [29]. Recently, Lee *et al.* developed a method to classify dental arch forms to ensure both goodness of fit and pragmatic clinical application [30]. In an attempt to correlate tooth size, but not TSD, with different arch forms, Haralabakis *et al.* concluded that smaller teeth were associated with “wide” or “pointed” maxillary arch forms and “flat” mandibular arch forms [31].

Few studies have explored the predominant arch forms and the prevalence of Bolton tooth-size discrepancy among Saudi patients. Thus, this study examines the arch form distribution in a sample of Saudi orthodontic patients, to evaluate the percentage of patients who present with a significant tooth-size discrepancy, and to investigate the possible association between arch form, clinically significant tooth-size discrepancy, and sagittal molar relationship.

## Methods

All available pretreatment orthodontic records of patients who attended the orthodontic clinics at the College of Dentistry, King Saud University, and a private orthodontic clinic in Riyadh, Saudi Arabia, were reviewed, and orthodontic casts from 230 patients matching the following selection criteria were included: Good-quality pretreatment study casts; fully erupted permanent teeth at least from first molar to first molar; absence of tooth crown size alteration (proximal restorations); no history of trauma or orthodontic treatment; and Saudi ethnicity. Ethical approval was obtained from the College of Dentistry Research Center (Registration No. NF 2271).

### Molar relation determination

Molar relationship (anteroposterior dental arch relationship) was assessed according to Angle’s definition. Molar Class I was defined as occurring where the mesiobuccal cusp of the upper first molar occluded with the mesiobuccal groove of the lower first molar, or within less than half a cusp width anteriorly or posteriorly. Mismatched right and left molar classifications were considered “asymmetric”.

### Arch-form analysis

Mandibular models were digitally scanned (Epson® Perfection V750-M Pro Scanner, Seiko Epson Corporation, Nagano, Japan) and a ruler was used for size calibration. The most facial aspect of 13 proximal contact areas around the arch was digitized using AutoCAD software (AutoCAD 2012, Autodesk, Inc., San Rafael, United States). The clinical bracket point for each tooth was located facially via a line perpendicular to that connecting the mesial and distal contact points of each tooth [22, 23, 32]. Then, tapered, ovoid, and square arch-form templates (3 M Unitek) were used to classify each case, based on the arch form that provided the best fit to the eight clinical bracket points ranging from the mandibular right first premolar to the left first premolar [33].

### Tooth-size measurement

A digital caliper was used to measure the mesiodistal crown diameters of all teeth (from first molar to first molar) to the nearest 0.01 mm [34]. The width of each tooth was measured from its mesial contact point to its distal contact point at its greatest mesiodistal width. Bolton’s formulae were used to calculate tooth-size ratios [16, 35]:

$$ \frac{{\displaystyle \sum}\mathrm{M}\mathrm{D}\ \mathrm{of}\ \mathrm{M}\mathrm{andibular}\ 3-3}{{\displaystyle \sum}\mathrm{M}\mathrm{D}\ \mathrm{of}\ \mathrm{M}\mathrm{axillary}\ 3-3}\times 100= Anterior\  Ratio\kern0.24em \left(\mathrm{Normal}\;\mathrm{value}=77.2\%\pm 1.65\right) $$

$$ \frac{{\displaystyle \sum}\mathrm{M}\mathrm{D}\ \mathrm{of}\ \mathrm{M}\mathrm{andibular}\ 6-6}{{\displaystyle \sum}\mathrm{M}\mathrm{D}\ \mathrm{of}\ \mathrm{M}\mathrm{axillary}\ 6-6}\times 100= Overall\  Ratio\kern0.24em \left(\mathrm{Normal}\ \mathrm{value} = 91.3\% \pm 1.91\right) $$

### Data analysis

Data were evaluated using PASW® Statistics 18 (SPSS Inc., Chicago, Illinois, United States), and the level of significance was set at p < 0.05. The following tests were used:

- Error of method: for intra-examiner reliability, measurements were compared via coefficient of reliability and kappa statistics. Within a two-week period, the mesiodistal widths of 10 pairs of casts were re-measured by the same investigator, and a high coefficient of reliability was observed (r = 0.936). Arch forms were re-determined by the same investigator for 19 lower casts and perfect agreement was observed between the first and second evaluations (kappa score of 1).
- Descriptive analysis including the prevalence of Bolton discrepancy and distribution of arch form types among the sample.
- Chi-square, *t*-test, and ANOVA were used to evaluate the presence of an association.

## Results

The demographic characteristics of the sample group and the distribution of sagittal molar classes and arch forms are shown in Table 1. No significant differences were observed between male and female patients in the distribution of molar relationships or arch forms (chi-square test). More than half of the cases were Class I, followed by asymmetric molar relationship, Class II, and then Class III. Arch forms were more equally distributed between the three shapes, and the most frequent form was the ovoid (p = 0.57). As shown in Table 2, no relationship was observed between the interarch relationships and the mandibular arch forms.

**Table 1:** Demographic data of the sample and the distribution of the molar classes and the arch forms

Age (year)	Mean ± SD	17.26 ± 6.47	Range	9.6-58.6
Gender	Male	109 (47.4 %)		
	Female	121 (52.6 %)		
		Male	Female	Total
Molar Classification	Class I	57 (24.8 %)	75 (32.6 %)	132 (57.4 %)
	Class II	15 (6.5 %)	15 (6.5 %)	30 (13 %)
	Class III	14 (6.1 %)	11 (4.8 %)	25 (10.9 %)
	Asymmetric	23 (10 %)	20 (8.7 %)	43 (18.7 %)
		p = 0.493*		
		Male	Female	Total
Arch Form	Square	27 (11.7 %)	37 (16.1 %)	64 (27.8 %)
	Ovoid	45 (19.6 %)	42 (18.3 %)	87 (37.8 %)
	Tapered	37 (16.1 %)	42 (18.3 %)	79 (34.3 %)
		p = 0.507*		

*Chi-square test: not statistically significant. Data presented as n (%)

**Table 2:** Distribution of arch forms across different sagittal molar relationship

Arch Form	Molar Classification				
	Class I	Class II	Class III	Asymmetric	Total
Square	37 (16.1 %)	8 (3.5 %)	6 (2.6 %)	13 (5.7 %)	64 (27.8 %)
Ovoid	50 (21.7 %)	12 (5.2 %)	10 (4.3 %)	15 (6.5 %)	87 (37.8 %)
Tapered	45 (19.6 %)	10 (4.3 %)	9 (3.9 %)	15 (6.5 %)	79 (34.3 %)
Total	132 (57.4 %)	30 (13.0 %)	25 (10.9 %)	43 (18.7 %)	230 (100 %)

Chi-square p-value = 0.998. Data presented as n (%)

**Fig. 1:**
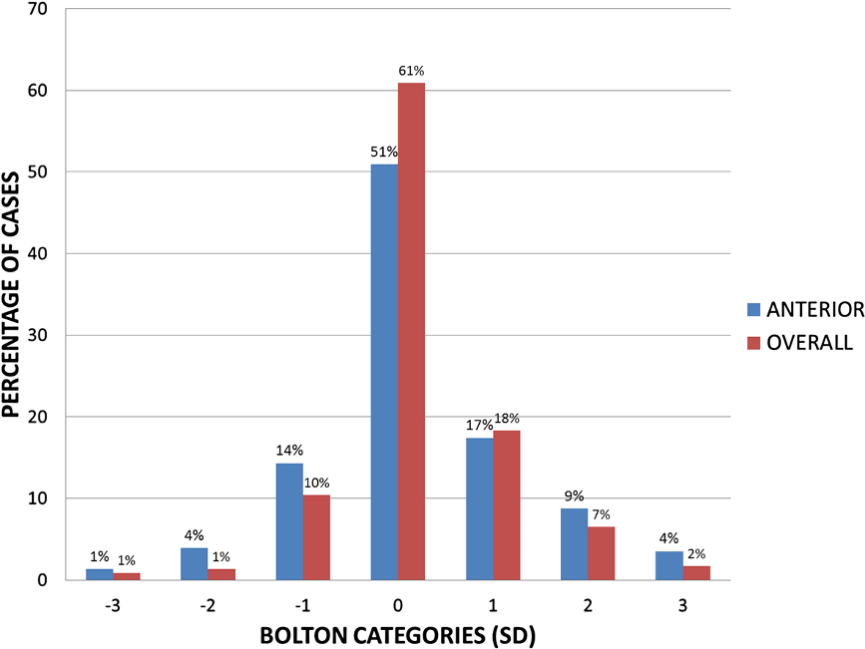
Frequency distribution of the cases with anterior and overall Bolton discrepancy: 0; No discrepancy (±1 SD), 1; 1–2 SD, 2; 2–3 SD, 3; >3 SD

Approximately half (49.1 %) of the sample showed an anterior Bolton tooth-size discrepancy i.e. exceeding ±1 standard deviation (SD) (<75.55 or >78.85), while only 39.1 % showed an overall Bolton discrepancy (<89.39 or >93.21) (Fig. 1). More cases showed high tooth-size ratio (29.6 % anterior and 26.5 % overall) than a low ratio (19.5 % anterior and 12.6 % overall).

In Bolton analysis, a significant discrepancy was evident when the tooth-size ratio exceeded two SDs from Bolton’s mean (<73.9 or >80.5 for the anterior ratio, <87.5 or >95.1 for the overall ratio) [35]. No association was observed between the presence of a significant Bolton discrepancy and the sagittal molar relationship or arch form (Table 3).

**Table 3:** Prevalence of tooth-size discrepancies of anterior and overall ratio defined as <2 SD or >2 SD by malocclusion, arch form, and gender and results of chi-square tests

	Anterior Bolton			Overall Bolton			
	±2SD	>2SD or <2SD	Chi-square test	±2SD	>2SD or <2SD	Chi-square test	Total
Class I	110 (47.8 %)	22 (9.6 %)	P = 0.966	119 (51.7 %)	13 (5.6 %)	P = 0.089	132 (57.4 %)
Class II	24 (10.4 %)	6 (2.6 %)		27 (11.7 %)	3 (1.3 %)		30 (13 %)
Class III	21 (9.1 %)	4 (1.7 %)		19 (8.3 %)	6 (2.6 %)		25 (10.9 %)
Asymmetric	35 (15.2 %)	8 (3.5 %)		41 (17.8 %)	2 (0.9 %)		43 (18.7 %)
Total	190 (82.6 %)	40 (17.4 %)		206 (89.6 %)	24 (10.4 %)		230 (100 %)
Square	50 (21.7 %)	15 (6.5 %)	P = 0.459	58 (25.2 %)	6 (2.6 %)	P = 0.910	64 (27.8 %)
Ovoid	72 (31.3 %)	14 (6.1 %)		77 (33.5 %)	10 (4.3 %)		87 (37.8 %)
Tapered	68 (29.6 %)	11 (4.8 %)		71 (30.9 %)	8 (3.5 %)		79 (34.3 %)
Total	190 (82.6 %)	40 (17.4 %)		206 (89.6 %)	24 (10.4 %)		230 (100 %)
Male	90 (39.1 %)	19 (8.3 %)	P = 0.988	93 (40.4 %)	16 (6.9 %)	P = 0.037*	109 (47.4 %)
Female	100 (43.5 %)	21 (9.1 %)		113 (49.1 %)	8 (3.5 %)		121 (52.6 %)
Total	190 (82.6 %)	40 (17.4 %)		206 (89.6 %)	24 (10.4 %)		230 (100 %)

*Statistically significant

ANOVA showed no significant difference in anterior ratio or overall ratio by sagittal molar class or arch form for the study sample as a whole. However, *t*-test results showed a significant difference in the prevalence of overall Bolton discrepancy between males (mean = 92.306) and females (mean = 91.545) (p = 0.013). No significant difference was observed in the anterior ratio between males (mean = 77.883) and females (mean = 77.329) (p = 0.08).

**Fig. 2:**
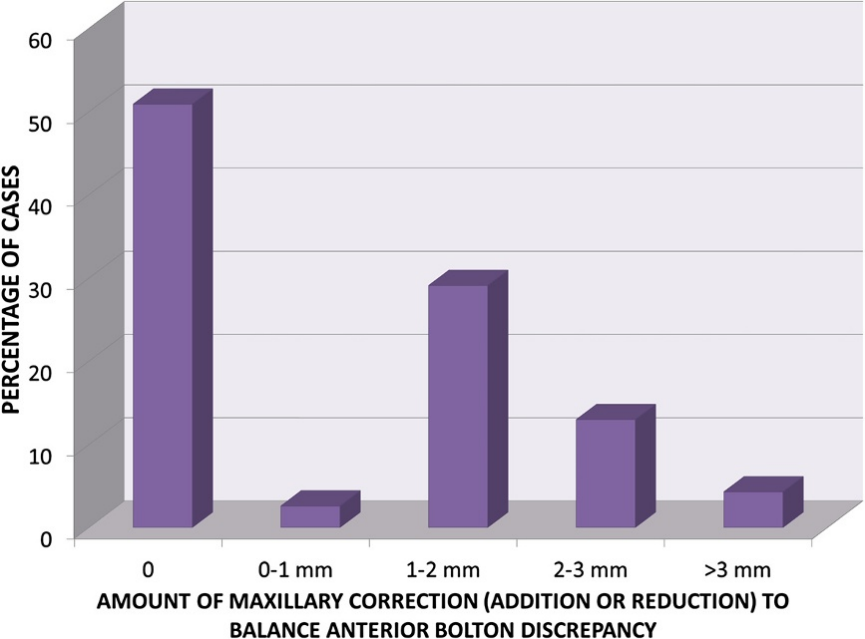
Frequency distribution of the cases defined by the amount of correction required to balance the anterior Bolton discrepancy in the maxillary anterior teeth

The distribution of the cases based on the amount of tooth-size correction required to balance the anterior Bolton discrepancy in the maxillary teeth (reduction or addition) is shown in Fig. 2.

## Discussion

Previous studies have reported significant differences in head form and arch form between various ethnic groups [22–24, 36–38]. Thus, careful selection of preformed archwires that match the patient’s original arch form and ethnicity is essential for a stable result.

Our Saudi group showed a significantly different distribution of arch forms compared with Egyptian, Caucasian [25], Israeli [24], Korean [23], and Japanese [22] populations (Table 4). Israeli orthodontic patients tend to have significantly more ovoid and fewer square arch-forms compared to the Saudi sample. Asian (Korean and Japanese) patients present with significantly more square and fewer tapered arch-forms than our sample. The Saudi group showed no relationship between Class III malocclusion and square arch form, contrary to previous findings in Egyptian, Caucasian, Israeli, Korean, and Japanese populations [22–25]. The natural compensation pattern of the mandibular anterior teeth characterized by lingual tipping and the resultant flattening of the anterior segment of the mandibular arch might explain the frequent observation of square arch form among Class III malocclusion cases in other populations. The small number of patients with Class III molar relationship in our sample, and the possibility that these cases present with mild Class III malocclusion lacking the usual developmental pattern of compensation might explain the lack of the square arch form in the study sample. In cases with Class II malocclusion, the distribution of the arch form was not different from that of the Class I group. This support the finding of Felton *et al.*, who reported little difference in arch forms between malocclusions groups [20]. However, other studies reported that Class II arches were more commonly associated with tapered arch forms and lower prevalence of the ovoid arch form in Caucasian subjects [22, 23]. Egyptian patients, on the other hand, showed an opposite tendency toward increased frequency of ovoid arch form in Class II malocclusion, a finding that supports the observation of ethnic variations in the distribution of arch forms [25].

**Table 4:** The distribution of arch forms between the Saudi sample and other ethnicities

Arch Form	Egyptian [25] (n = 94)	Caucasian [25] (n = 92)	Israeli [24] (n = 134)	Korean [23] (n = 368)	Japanese [22] (n = 160)	Saudi Present Study (n = 230)
Square	29 (31 %)	19 (21 %)	23 (17.2 %)	172 (46.7 %)	73 (45.6 %)	64 (27.8 %)
Ovoid	37 (39 %)	39 (42 %)	68 (50.7 %)	127 (34.5 %)	68 (42.5 %)	87 (37.8 %)
Tapered	28 (30 %)	34 (37 %)	43 (32.1 %)	69 (18.8 %)	19 (11.9 %)	79 (34.3 %)
p-value	0.714	0.409	0.024*	<0.0001*	<0.0001*	

*Chi-square test: statistically significant. Data presented as n (%)

Similarly to previous studies, our group showed no sex differences in arch form [21, 25, 39–42]. However, some studies reported that some male arches are larger than female arches [25, 31]. The current trend in clinical practice has shifted towards the utilization of digital models that are able to capture the details of the facial surfaces via three-dimensional imaging. New developments in software technology should enable clinicians to more accurately identify arch forms appropriate for each patient and to select custom-fit archwires.

A large proportion of orthodontic patients present with tooth-size discrepancy. Those who have anterior or overall ratios beyond 2 SDs are considered to have a significant Bolton discrepancy. In the Saudi sample, 17.4 % of patients had significant anterior tooth-size discrepancy. This figure matches the findings for a British orthodontic population (17.4 %) and a Croatian population (16.28 %) but was lower than the prevalence in a Turkish population reported by Uysal and Sari (21.3 %), Dominican American population reported by Santoro *et al.* (28 %), and American population reported by Freeman *et al.* (30.6 %) [9, 17, 43–45]. The finding that approximately one-fifth of orthodontic patients present with a significant tooth-size discrepancy clearly highlights the need to conduct Bolton analysis as an essential part of the initial work-up of any orthodontic case. Early identification of such a discrepancy assists clinicians in planning appropriate treatment method (enamel interproximal reduction or composite resin bonding/veneers), facilitates discussion of the treatment plan with the patient, and improves communication with other dental specialists [46].

In the present study, sagittal molar classification was not related to the distribution of the tooth-size discrepancy groups. This was in agreement with the findings of Uysal and Sari, who reported no difference in tooth-size ratios between malocclusion groups in a Turkish population, the findings of Crosby and Alexander in an American population, and O’Mahony *et al.* in an Irish population [17, 47, 48]. However, Araujo and Souki reported that Brazilian individuals with Angle Class I and Class III malocclusions showed significantly higher prevalence of tooth-size discrepancies than individuals with Class II malocclusions [4]. In a Chinese population, the trend towards higher tooth-size ratios in Class III malocclusion was noted by Nie and Lin and by Ta *et al.* [3, 5]. This trend was also reported by Alkofide and Hashim, and by Strujic *et al.* in Saudi population and Croatian populations respectively [6, 45]. In the present study, approximately one-third of Class III cases presented with significant tooth-size discrepancy. This tendency was non-significant, which might be attributed to the small proportion of individuals with Class III malocclusion in the present study. This study is the first to report asymmetric molar relationship as a separate category in an attempt to identify the possible contribution of tooth-size discrepancy to the etiology of such malocclusion. No relationship was detected between the presence of Bolton discrepancy and the asymmetric molar relationship, which may indicate that the known possible explanations for this classification (skeletal asymmetry, centric relation-centric occlusion shift, dental asymmetry due to drifting or dental anomalies) remain the underlying causes. The current assessment of sagittal malocclusion is limited by the fact that sagittal molar relationship is insufficient for diagnosis of Class II or Class III malocclusion, and because other sagittal variables such as overjet and skeletal sagittal discrepancy were not evaluated in this study. Moreover, the sagittal molar relationship can be altered by molar mesial migration.

Most prior studies reported no significant differences in anterior or overall tooth-size ratio between males and females [2, 3, 6, 8, 15, 43, 48]. In the present study, overall Bolton discrepancy was significantly more prevalent among males than females. However, in a Turkish population, Uysal et al. reported that males showed significantly lower overall ratio than females [7, 17]. These conflicting results for tooth sizes may be explained by the differing ethnicities of the study groups.

Arch form types were not related to the presence of tooth-size discrepancy. Therefore, arch form is likely determined by patient-specific genetic and environmental factors, and orthodontists need to recognize the uniqueness of each case in their treatment planning.

## Conclusions

Based on the results of the present study, the following conclusions can be drawn:

- In Saudis, there were more ovoid cases forms than tapered and square but no single arch form was significantly more common.
- Arch form types were not associated with gender, sagittal molar relationship, or the presence of tooth-size discrepancy.
- Sexual dimorphism was evident in the prevalence of overall Bolton tooth-size discrepancy.

## Abbreviations

TSD: Tooth size discrepancy
PASW®: Predictive analytics software
ANOVA: Analysis of variance
SD: Standard deviation
N: Number

## References

[1] Proffit WR (2006). Contemporary Orthodontics.

[2] Smith SS, Buschang PH, Watanabe E (2000). Interarch tooth size relationships of 3 populations: “does Bolton’s analysis apply?”. Am J Orthod Dentofacial Orthop.

[3] Nie Q, Lin J (1999). Comparison of intermaxillary tooth size discrepancies among different malocclusion groups. Am J Orthod Dentofacial Orthop.

[4] Araujo E, Souki M (2003). Bolton anterior tooth size discrepancies among different malocclusion groups. Angle Orthod.

[5] Ta TA, Ling JY, Hagg U (2001). Tooth-size discrepancies among different occlusion groups of southern Chinese children. Am J Orthod Dentofacial Orthop.

[6] Alkofide E, Hashim H (2002). Intermaxillary tooth size discrepancies among different malocclusion classes: a comparative study. J Clin Pediatr Dent.

[7] Uysal T, Sari Z, Basciftci FA, Memili B (2005). Intermaxillary tooth size discrepancy and malocclusion: is there a relation?. Angle Orthod.

[8] Lavelle CL (1972). Maxillary and mandibular tooth size in different racial groups and in different occlusal categories. Am J Orthod.

[9] Santoro M, Ayoub ME, Pardi VA, Cangialosi TJ (2000). Mesiodistal crown dimensions and tooth size discrepancy of the permanent dentition of Dominican Americans. Angle Orthod.

[10] Sameshima GT (2006). Bolton tooth size variation among four ethnic groups. 84th IADR General Session & Exhibition. Brisbane.

[11] Akyalcin S, Dogan S, Dincer B, Erdinc AM, Oncag G (2006). Bolton tooth size discrepancies in skeletal Class I individuals presenting with different dental angle classifications. Angle Orthod.

[12] Basaran G, Selek M, Hamamci O, Akkus Z (2006). Intermaxillary Bolton tooth size discrepancies among different malocclusion groups. Angle Orthod.

[13] Al-Tamimi T, Hashim HA (2005). Bolton tooth-size ratio revisited. World J Orthod.

[14] Endo T, Shundo I, Abe R, Ishida K, Yoshino S, Shimooka S (2007). Applicability of Bolton’s tooth size ratios to a Japanese orthodontic population. Odontology.

[15] Johe RS, Steinhart T, Sado N, Greenberg B, Jing S (2010). Intermaxillary tooth-size discrepancies in different sexes, malocclusion groups, and ethnicities. Am J Orthod Dentofacial Orthop.

[16] Bolton WA (1958). Disharmony in tooth size and its relation to the analysis and treatment of malocclusion. Angle Orthod.

[17] Uysal T, Sari Z (2005). Intermaxillary tooth size discrepancy and mesiodistal crown dimensions for a Turkish population. Am J Orthod Dentofacial Orthop.

[18] Hickey J, Zarb G, Bolender C (1985). Boucher’s prosthodontic treatment for edentulous patients.

[19] Little RM, Wallen TR, Riedel RA (1981). Stability and relapse of mandibular anterior alignment-first premolar extraction cases treated by traditional edgewise orthodontics. Am J Orthod.

[20] Felton JM, Sinclair PM, Jones DL, Alexander RG (1987). A computerized analysis of the shape and stability of mandibular arch form. Am J Orthod Dentofacial Orthop.

[21] Raberin M, Laumon B, Martin JL, Brunner F (1993). Dimensions and form of dental arches in subjects with normal occlusions. Am J Orthod Dentofacial Orthop.

[22] Nojima K, McLaughlin RP, Isshiki Y, Sinclair PM (2001). A comparative study of Caucasian and Japanese mandibular clinical arch forms. Angle Orthod.

[23] Kook YA, Nojima K, Moon HB, McLaughlin RP, Sinclair PM (2004). Comparison of arch forms between Korean and North American white populations. Am J Orthod Dentofacial Orthop.

[24] Gafni Y, Tzur-Gadassi L, Nojima K, McLaughlin RP, Abed Y, Redlich M (2011). Comparison of arch forms between Israeli and North American white populations. Am J Orthod Dentofacial Orthop.

[25] Bayome M, Sameshima GT, Kim Y, Nojima K, Baek SH, Kook YA (2011). Comparison of arch forms between Egyptian and North American white populations. Am J Orthod Dentofacial Orthop.

[26] Taner TU, Ciger S, El H, Germec D, Es A (2004). Evaluation of dental arch width and form changes after orthodontic treatment and retention with a new computerized method. Am J Orthod Dentofacial Orthop.

[27] Trivino T, Siqueira DF, Scanavini MA (2008). A new concept of mandibular dental arch forms with normal occlusion. Am J Orthod Dentofacial Orthop.

[28] Oda S, Arai K, Nakahara R (2010). Commercially available archwire forms compared with normal dental arch forms in a Japanese population. Am J Orthod Dentofacial Orthop.

[29] Arai K, Will LA (2011). Subjective classification and objective analysis of the mandibular dental-arch form of orthodontic patients. Am J Orthod Dentofacial Orthop.

[30] Lee SJ, Lee S, Lim J, Park HJ, Wheeler TT (2011). Method to classify dental arch forms. Am J Orthod Dentofacial Orthop.

[31] Haralabakis NB, Sifakakis I, Papagrigorakis M, Papadakis G (2006). The correlation of sexual dimorphism in tooth size and arch form. World J Orthod.

[32] Andrews L (1989). Straight wire—the concept and appliance.

[33] Battagel JM (1996). Individualized catenary curves: their relationship to arch form and perimeter. Br J Orthod.

[34] Jensen E, Kai-Jen Yen P, Moorrees CF, Thomsen SO (1957). Mesiodistal crown diameters of the deciduous and permanent teeth in individuals. J Dent Res.

[35] Bolton WA (1962). The clinical application of a tooth-size analysis. Am J Orthod.

[36] Koyoumdjisky-Kaye E, Zilberman Y, Zeevi Z (1976). A comparative study of tooth and dental arch dimensions in Jewish children of different ethnic descent. I. Kurds and Yemenites. Am J Phys Anthropol.

[37] Enlow DH (1990). Handbook of facial growth.

[38] Kasai K, Kanazawa E, Aboshi H, Richards LC, Matsuno M (1997). Dental arch form in three Pacific populations: a comparison with Japanese and Australian aboriginal samples. J Nihon University School of Dentistry.

[39] DeKock WH (1972). Dental arch depth and width studied longitudinally from 12 years of age to adulthood. Am J Orthod.

[40] Ferrario VF, Sforza C, Miani A, Tartaglia G (1994). Mathematical definition of the shape of dental arches in human permanent healthy dentitions. Eur J Orthod.

[41] Collins BP, Harris EF (1998). Arch form in American blacks and whites with malocclusions. J Tennessee Dental Association.

[42] Hassanali J, Amwayi P (1993). Biometric analysis of the dental casts of Maasai following traditional extraction of mandibular permanent central incisors and of Kikuyu children. Eur J Orthod.

[43] Othman SA, Harradine NW (2007). Tooth-size discrepancy and Bolton’s ratios: the reproducibility and speed of two methods of measurement. J Orthod.

[44] Freeman JE, Maskeroni AJ, Lorton L (1996). Frequency of Bolton tooth-size discrepancies among orthodontic patients. Am J Orthod Dentofacial Orthop.

[45] Strujic M, Anic-Milosevic S, Mestrovic S, Slaj M (2009). Tooth size discrepancy in orthodontic patients among different malocclusion groups. Eur J Orthod.

[46] Tong H, Chen D, Xu L, Liu P (2004). The effect of premolar extractions on tooth size discrepancies. Angle Orthod.

[47] Crosby DR, Alexander CG (1989). The occurrence of tooth size discrepancies among different malocclusion groups. Am J Orthod Dentofacial Orthop.

[48] O’Mahony G, Millett DT, Barry MK, McIntyre GT, Cronin MS (2011). Tooth size discrepancies in Irish orthodontic patients among different malocclusion groups. Angle Orthod.

